# Visual Behaviours of Expert Padel Athletes When Playing on Court: An In Situ Approach with a Portable Eye Tracker

**DOI:** 10.3390/s23031438

**Published:** 2023-01-28

**Authors:** Carlos Espino Palma, Vicente Luis del Campo, Diego Muñoz Marín

**Affiliations:** Faculty of Sport Sciences, University of Extremadura, Avda. de la Universidad, s/n, 10003 Caceres, Spain

**Keywords:** eye-tracking, gaze behaviour, real-world scenarios, padel

## Abstract

Eye-tracking research has allowed the characterisation of gaze behaviours in some racket sports (e.g., tennis, badminton), both in controlled laboratory settings and in real-world scenarios. However, there are no studies about visual patterns displayed by athletes in padel. Method: The aim of this exploratory case study was to address the visual behaviours of eight young expert padel athletes when playing match games on a padel court. Specifically, their gaze behaviours were examined with an in situ approach while returned trays/smashes, serves, and volleys were performed by their counterparts. Gaze patterns were registered with an SMI Eye Tracking Glasses 2 Wireless. Results: The participants’ gaze was mainly focused on the ball-flight trajectory and on the upper body of the opponents because they were the two visual locations with a larger number of fixations and longer fixation time. No differences were found in these variables for each type of visual location when the three return situations were compared, or independently of them. Conclusions: Padel players displayed a similar gaze behaviour during different representative return situations. This visual pattern was characterised by fixating at the ball and some opponents’ upper kinematics (head, shoulders, trunk, and the region of arm–hand–racket) to perform real interceptive actions while playing against them on a padel court.

## 1. Introduction

Eye-tracking technology has been used to analyse visual behaviours of athletes when observing specific actions pertaining to racket sports, for instance, in badminton [1,2], table tennis [3,4] and tennis [5,6,7,8]. This technology has provided an opportunity for researchers to specify what visual information athletes use at different levels of expertise to intercept fast balls/shuttlecocks and/or anticipate the opponents’ actions. Therefore, eye-tracking technology allows an exploration of the underlying mechanisms of time cognitive processing in expert performance [9]). According to this author, there are three types of eye trackers based on a video-based pupil and corneal reflection system: table/desk-mounted, head-mounted, and remote systems.

Eye trackers provide measures of eye movements and mapping of the gaze to the ‘real world’ [10]. Eye-tracking systems also allow some insights about the visual attention of athletes, throughout the analysis of gaze direction, because the location of the gaze and the orientation of the attention are closely related [11]. The first studies using eye-tracking technology in the sport domain occurred in the early 1980s/1990s (e.g., [7,12,13,14,15], although the first one dates back to 1976 with a comparison between the gaze behaviours of expert and novice athletes in basketball [16].

Most studies using eye-tracking technology with expert players have been carried in controlled laboratory settings (69% vs. the 31% studies performed in situ; see [17]. However, there are some doubts about the generalisation of results from the lab to real-world environments because athletes are usually required to remain in a static position during the experiment with verbal and/or simplified responses [18]. To guarantee the ecological validity and representative designs for studies on perceptual–cognitive skills, the use of mobile (head-mounted) eye-tracking systems to measure the gaze behaviours of athletes outside of the laboratory (i.e., in a naturalistic environment as the playing field), but ensuring reliability of measurements with a minimum level of experimental control [17], should be beneficial.

A relevant mechanism underlying high levels of motor performance has been the efficient use of visual information available in dynamic and changing environments. For example, expert athletes usually observe the sport environment with an economical gaze pattern characterised by fewer fixations of longer duration, compared to other groups of low-skill levels [19]. Therefore, experts seem to be better at selectively allocating their attention and ignoring irrelevant areas. As a result, skilled players move their focus from one area of interest to another, guided by their visuomotor experiences and/or previous knowledge, which is in line with the guided search theory [20]. This observation supports the information-reduction hypothesis that explains the ability of expert athletes to allocate visual attention to the most relevant stimuli of the environment [21]. For example, in tennis, high-skill-level players were better than their less-skilled counterparts at picking advanced cues from their opponent’s kinematics [8,22,23,24].

The majority of tennis studies including measures of gaze patterns has been focused on the use of anticipatory cues for the serve return because it is a high-pressure-time situation in this sport [5,7,12]. Specifically, these studies addressed the differences in visual behaviours between tennis players with different skill levels when observing servers’ kinematics (i.e., information from the body orientation of an opponent; see [24]. Thus, these studies have been centred mainly on laboratory-based designs [25]. For example, Lin et al., [26] concluded that expert tennis players showed a visual pattern mainly focused on the upper body and the ball in returning a serve, while novice players showed a more distributed visual strategy with longer fixations outside of the server’s body. Similarly, Rosker and Majcen [6] stated that international tennis players showed an anticipatory visual behaviour during the tossing hand movement and also better picking up of visual information during the final phases of the server’ stroke, compared to other groups of national players. An exception was Singer et al. [27], who found that expert tennis players with better ranking displayed a smooth tracking of the ball after the serve, compared to lower-ranked players, when collecting visual behaviours of participants in situ (i.e., on the tennis court with movement responses). Luis-del Campo et al. [28] compared the visual and motor behaviours of novice tennis players when observing passing shots performed by an expert tennis player located at the back line of the court, both in laboratory and on court. These tennis players developed a differentiated visual behaviour depending on the experimental setting (i.e., they fixated more on the trunk and hip in laboratory and more on the head and shoulders on a court).

Padel is a modern doubles racket sport with current international presence in more than 60 countries [29]. According to the IPF, two pairs of players face each other following a tennis scoring system and similar rules to tennis (e.g., position of players, sides, serve, and returns). However, this sport is differentiated from tennis because it is played in an enclosed synthetic glass and metal court, allowing the ball to bounce on lateral and back walls for rallies. Their court dimensions are smaller compared to those of tennis. Based on these features, padel could be defined as an interceptive sport because it implies coordination between an athlete’s body, a held implement, opponents’ actions, and an object in the environment, similarly to squash, badminton, and tennis [30].

The existing literature for padel has been focused on match analysis to determine performance outcome measures [31,32,33,34], e.g., the age and gender [35], effectiveness at the net [36], effect of the return of serve on the server pair’s movement parameters [37], serve and serve-return strategies [38], length of the rally [39], efficacy of the lob [40] or the smash [41].

Altogether, the existing literature shows that despite the technological advances in the development of mobile eye-tracking systems, laboratory research is still the most common methodology used to study gaze patterns in racket sports. As a result, there are scarce studies measuring the visual behaviours of athletes during “real life” situations. Therefore, there is a lack of studies investigating, in controlled laboratory settings or real-world situations, what visual information would be perceived by padel players when responding to different video projections containing specific movements of their counterparts or when they interact against them in situ during on-court situations.

This exploratory study aimed to evaluate the visual behaviours of expert padel players, using eye-tracking technology as a direct measurement method in sports-related research on perceptual–cognitive skills. The specific contribution of this study was to address the visual behaviours of padel players while observing real-life opponents’ shots. Therefore, it provides the first evidence about visual demands of these athletes when played on court (i.e., where did the padel players focus their gaze on?). Along this line, the study acquired gaze data under unrestricted test conditions because the players performed naturalistic responses (i.e., real-world movements) using a mobile eye-tracker that ensured the ecological validity of the experimental condition.

Specifically, we describe the visual search strategies performed by expert padel players when playing different match games on court. To embrace this endeavour, we used a head-mounted eye tracker to measure the natural gaze of these players while they performed specific movements without restrictions of mobility, but also with an experimental condition of full wireless control for observation of their gaze recordings. Additionally, we explored the set of visual fixations displayed by these participants in situ when faced with different representative padel situations, such as returns of counterparts’ trays/smashes, volleys, and serves. This information collected by a portable eye-tracking device could reveal some of the attentional demands that players may have while playing in “real life” situations.

## 2. Material and Methods

### 2.1. Ethical Approval

Participants voluntarily took part in the study, and their parents provided written informed consent prior to commencement because the participants were under 18 years at the beginning of the study. The research was performed according to the ethical standards of research of the University in accordance with the Declaration of Helsinki. Specifically, this study received approval from the Bioethics and Biosecurity Committee on 6 March 2018 (nº 33/2018). Participants and their legal tutors/parents received general information about the research contexts and signed an informed consent form (legal tutors/parents) but were naïve to the specific hypotheses.

### 2.2. Participants

We recruited 8 male padel players (aged 14–16; *M* = 14.5). We measured their height (*M* = 171.63 cm, *SD* = 5.32) and weight (*M* = 61.38 kg, *SD* = 6.97). All participants had a licence for padel competitions. They had accumulated a mean experience of training in padel for 5.75 years (*SD* = 0.89). The frequency of training was 3–4 days per week, lasting 1 h and 30 min by day.

The inclusion criteria were playing in official competitions at a national level and being one of the players pertaining to the U-16 padel team of one Regional Padel Federation in Spain. According to [42], the athletes of this study were considered experts because they had competed at the maximal level of competition for this sport in the country and participated in different national padel tournaments within their category. Exclusion criteria included being injured and wearing eyeglasses. All participants used their own personal padel rackets and wore usual sportswear to ensure familiarity in performing tasks.

### 2.3. Instruments

**Figure 1:**
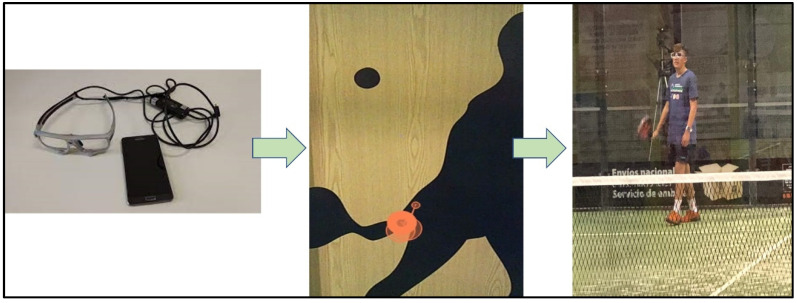
The SMI ETG 2w eye tracker used in this study: with a customised Samsung Galaxy S4 smart recorder (**left**), during the calibration process onto a wall sticker padel player (**centre**), and already working to capture a padel player’s natural gaze behaviour in real time (**right**).

An SMI Eye Tracking Glasses 2 Wireless (SMI ETG 2w) captured the padel players’ natural gaze behaviour in real time (Figure 1). This device is a non-invasive video-based glasses-type eye tracker with a sampling rate of 60 Hz binocular and a gaze-tracking accuracy of 0.5° over all distances. The design of the head gear ensured maximal peripheral perception and binocular vision according to the technical data provided by SensoMotoric Instruments GmbH© (2014).

The easy calibration setup of the SMI ETG 2w provided the opportunity to start recordings of padel players’ gaze within seconds because this process was performed near to the court. We also used a small smartphone recording unit to register fixations of all playing matches while ensuring a complete mobility of the participants when played these games. The ETG 2w smart recorder was controlled via wi-fi from a laptop. The SMI BeGaze analysis software allowed further analysis and reporting of eye-tracking data and scene videos. Specifically, we analysed the visual fixations of participants when playing match games.

A visual fixation was coded when the gaze remained within one degree of visual angle of a location for a minimum duration of at least 100 ms [43]. We utilised similar areas of interests for this study as in previous tennis studies [26,27] because padel and tennis are interceptive racket sports and share similar rules regarding position of players, sides, serve, returns, and scoring [32]. These visual fixations were upper body (head, shoulders, trunk, and the region of arm–hand–racket), lower body (hip and legs), ball trajectory, and other unclassified visual locations (i.e., those fixations that did not fall within any of the previous areas; for instance, net, court, or background).

We analysed the number of fixations and fixation duration times for the above fixation locations in each return situation. These values were expressed in absolute (e.g., total number of fixations and total duration of the fixations in each visual location), but also in relative measures (e.g., percentage of visual fixation: the number of fixations in each location divided by the total number of fixations; percentage of fixation time: the time fixating at each visual location divided by the total fixation time). We included these last relative measures because the number and duration of return situations were different in each participant and match game.

### 2.4. Procedures

The study was performed in an indoor official padel court, using artificial light to favour the working of the eye-tracking system. Upon participants’ arrival to the padel court, researchers provided them with information about the objectives of the study and then calibrated the eye-tracking device.

We used a case study design because it is a suitable strategy to bridge the science–practice gap [44] and because these types of designs attempt to understand how outcomes are achieved within applied practice [45]. Specifically, our cases study design was carried out on court to better address the visual behaviours of a small sample of padel players in natural interceptive tasks because the visual–motor actions in coincidence timing emerge from interactions between perceptual information and motor control [46]. Therefore, this experimental setup preserved the perception–action links in naturally coupled movement tasks as returns of shots hit by their counterparts.

The number of participants was similar to that in previous tennis studies when visual behaviours were recorded in situ (e.g., three participants in [27]; seven participants in [26]; or eight skilled players in [24]). We decided to analyse the visual behaviours of players when returning opponents’ shots of serve, volley, and tray/smash because these specific situations impose a high time pressure and, therefore, fast reactions due to the high speed of the ball and the closeness of the opponents. Each participant was recorded during two games of the match. As a result, a total of 128 fixations corresponding to all participants were analysed with the corresponding software of the portable eye tracker. For type of return situations, 51 fixations were identified while returning counterparts’ serves, 44 fixations while returning the volleys, and 32 fixations during returns of trays/smashes.

### 2.5. Statistical Analysis

To characterise the point-of-gaze data for the participants of this study, descriptive statistics of sums, means, and standard deviations were used to explore their visual patterns with respect to number of visual fixations made, fixation duration times, percentage of visual fixations, and percentage of fixation duration times on the specific visual locations described in the Instruments section. These values were calculated independently and according to each return situation. We used the type of return situation as the independent variable to address whether the participants developed a differentiated visual strategy in returning different shots performed by their counterparts (Level 1: tray/smash returns (Video S1); Level 2: serve returns (Video S2); Level 3: volley returns (Video S3)).

Shapiro–Wilk analysis confirmed that the data for most dependent variables did not display a normal distribution. Thus, the low number of padel players participating in the study, based on the rationale for a case study design, compelled us to perform nonparametric tests to test the influence of independent variables in the gaze variables. Specifically, the Kruskal–Wallis test was used to determine differences in these variables between types of return situations. Statistical analyses were performed using the statistical package SPSS v25.0 (© IBM Corporation, Armonk, NY, USA).

## 3. Results

Firstly, the padel players displayed a total of 128 fixations during the recorded playing games, with a duration fixation time of 138,377 milliseconds (ms). For areas of interest, the players engaged in 7 fixations on the upper body with a fixation time of 6545 ms, 9 fixations with a fixation time of 5799 ms (lower body), 109 fixations with 122,305 ms of fixation time (ball), and 3 fixations with 3728 ms of fixation time for the other unclassified visual locations.

**Figure 2:**
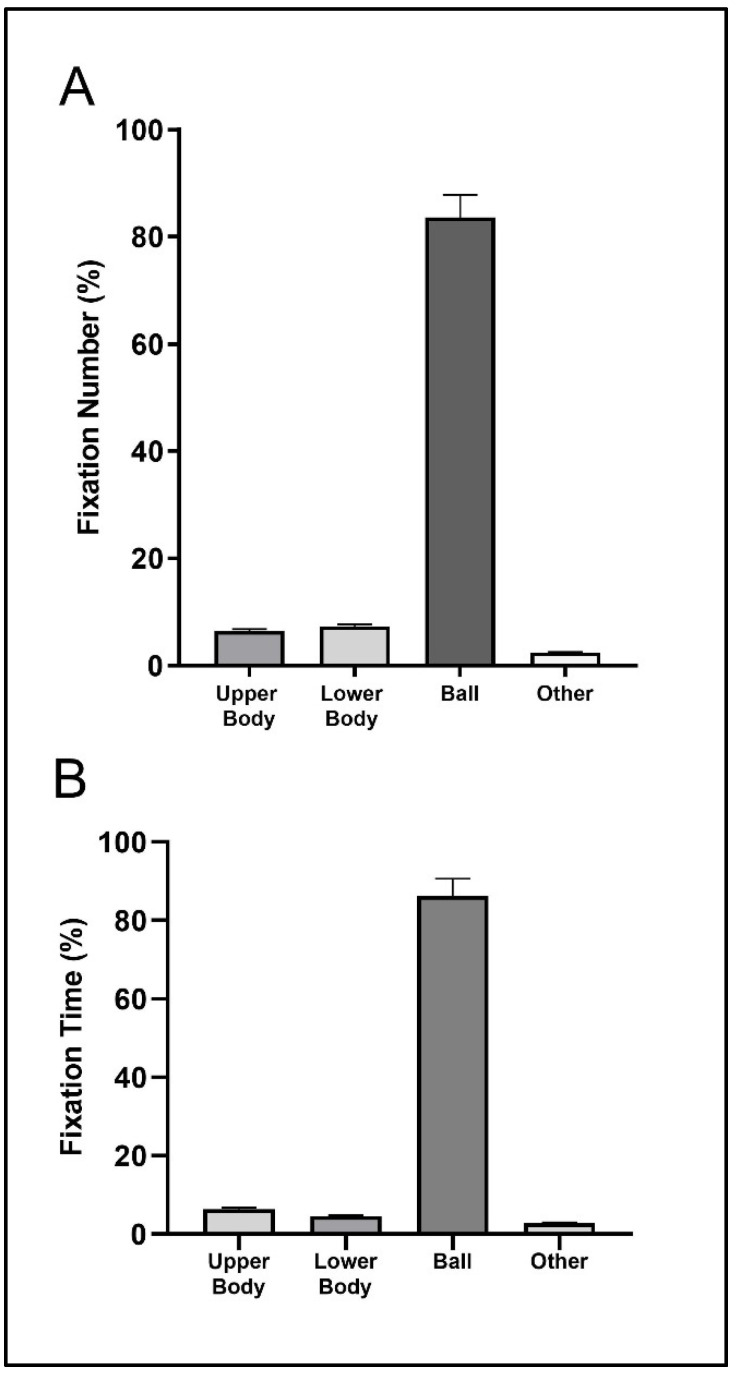
Mean (bar) and standard deviation (error bars with 95% confidence interval) of the relative fixation number (**A**) and fixation time (**B**) for the three return situations.

Figure 2 shows that the highest percentage of fixation corresponded to the ball location, with values close to 80% (fixation number) and 90% (fixation time) of the total number and time of fixations. The second visual location with the highest percentage of number and time of fixations was the lower body and upper body, with values near to the 5% of the total number and time of fixations.

**Figure 3:**
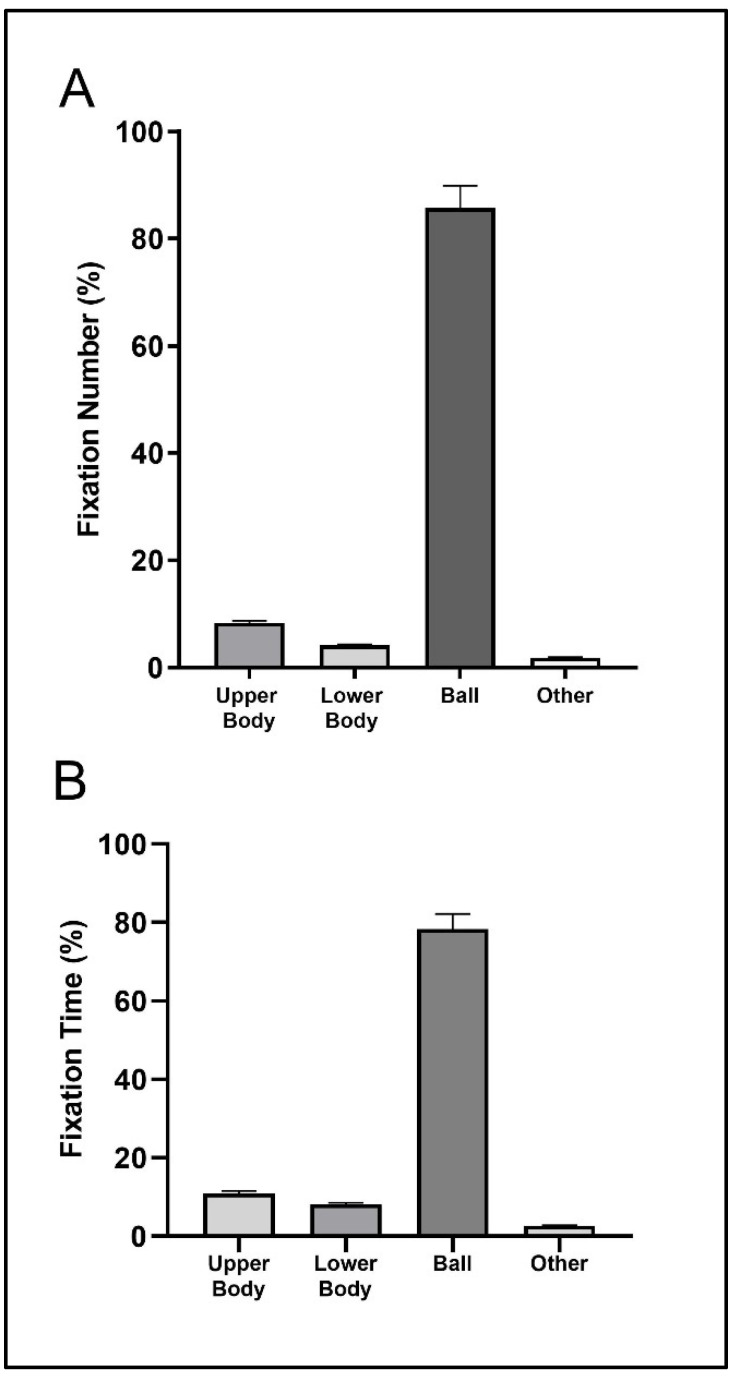
Mean (bar) and standard deviation (error bars with 95% confidence interval) of the relative fixation number (**A**) and fixation time (**B**) for the tray/smash return situation.

For the tray/smash return situation, the players engaged in 1 fixation with a duration of 1558 ms (upper body), 1 fixation with 994 ms of fixation time (lower body), 29 fixations with 34,438 ms of fixation time (ball), and 1 fixation with 1607 ms fixating at other unclassified visual locations. Figure 3 shows that the highest percentage was found in the ball location with values slightly higher (fixation number) and lower (fixation time) than 80% of the total number and time of fixations. The second visual location with the highest percentages of number and time of fixations was the upper body, with values near to 10% of the total number and time of fixations.

For the serve return situation, players engaged in 4 fixations with a duration of 3313 ms (upper body), 7 fixations with 4673 ms of fixation time (lower body), 38 fixations with 48,468 ms of fixation time (ball), and 2 fixations with 2121 ms fixating at other unclassified visual locations.

**Figure 4:**
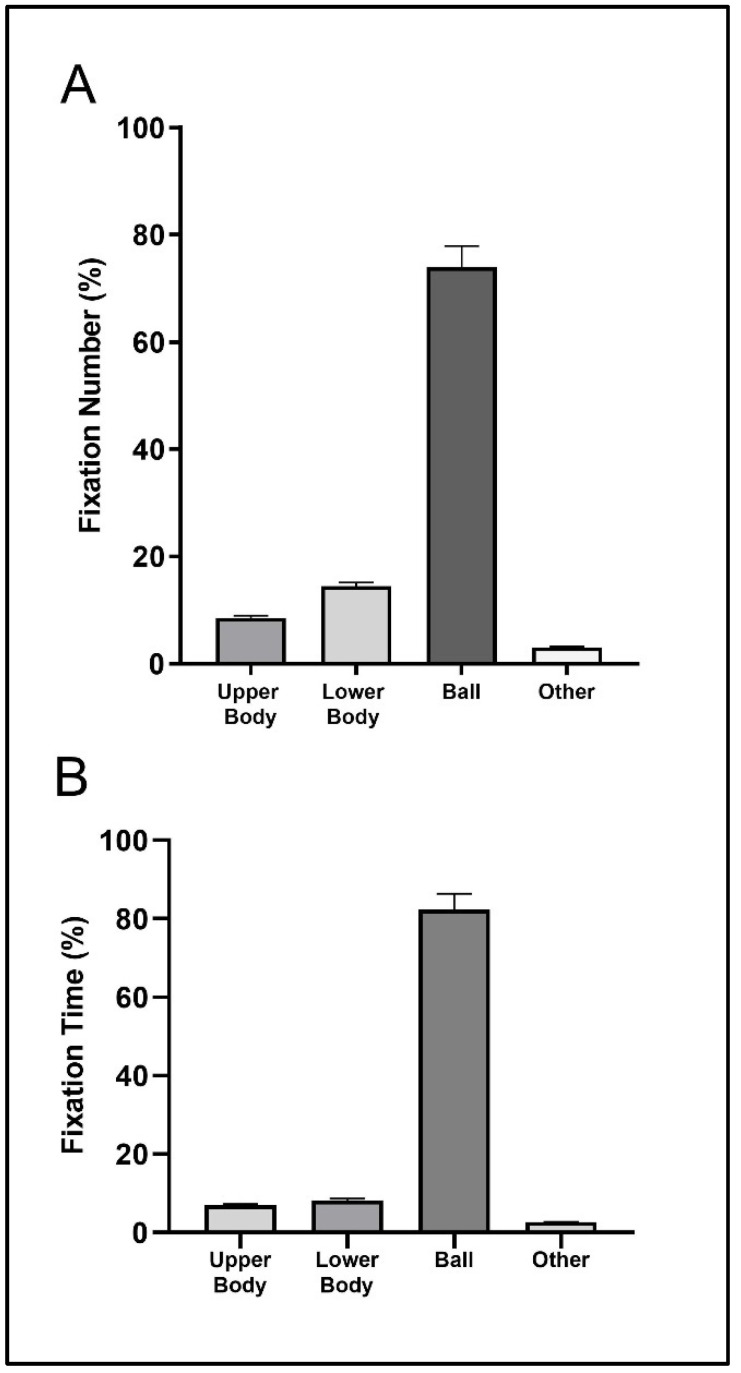
Mean (bar) and standard deviation (error bars with 95% confidence interval) of the relative fixation number (**A**) and fixation time (**B**) for the serve return situation.

Figure 4 shows that the highest percentage was found in the ball location with values slightly higher than 70% (fixation number) and 80% (fixation time) of the total number and time of fixations. The second visual location with the highest percentages of number and time of fixations was the lower body, with values slightly higher (fixation number) and lower (fixation time) than 10% of the total number and time of fixations.

**Figure 5:**
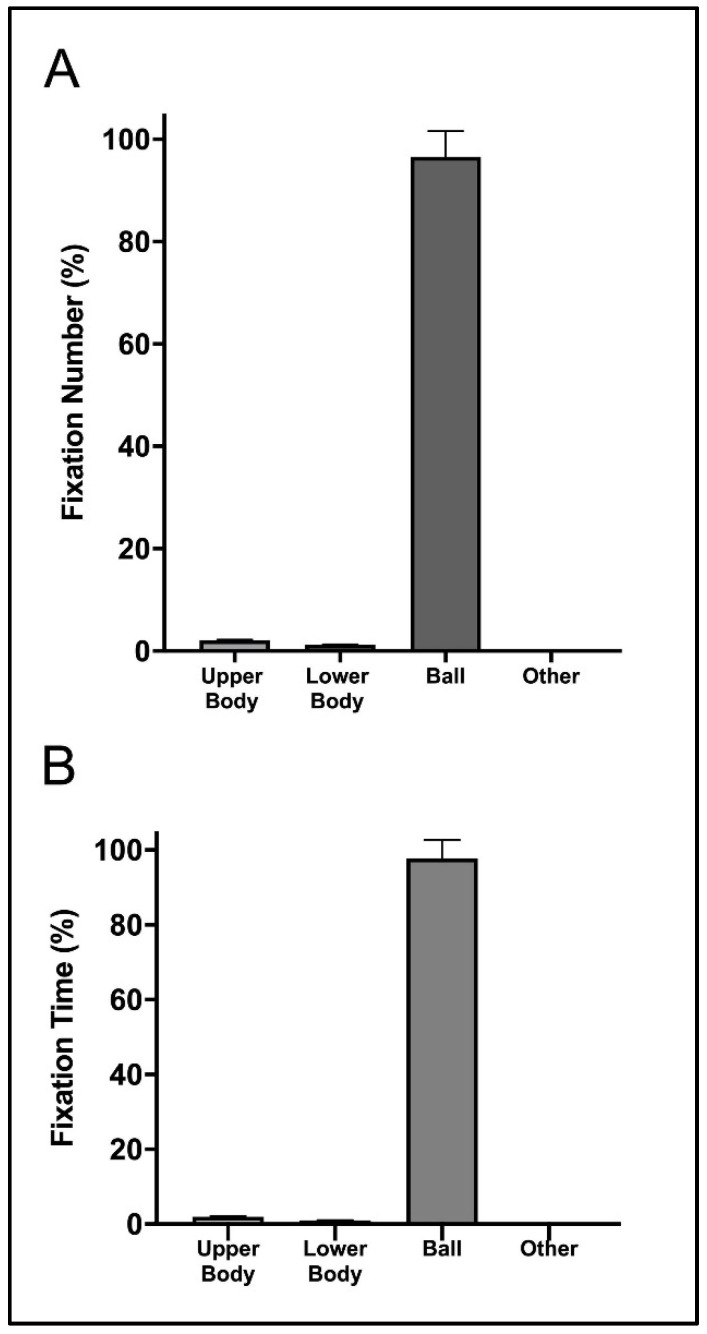
Mean (bar) and standard deviation (error bars with 95% confidence interval) of the relative fixation number (**A**) and fixation time (**B**) for the volley return situation.

For the volley return situation, the players engaged in 2 fixations with a duration of 1674 ms (upper body), 1 fixation with 132 ms of fixation time (lower body), 42 fixations with 39,399 ms of fixation time (ball), and no fixations at other unclassified visual locations. Figure 5 shows that the highest percentage was found in the ball location with values slightly higher than 90% of the total number and time of fixations, for both fixation number and fixation time variables. The second visual location with the largest percentage of total number and time of fixations was the upper body, with residual values of 2% of the total number and time of fixations, again for the fixation number and fixation time variables.

Finally, there were no differences in the variables of the study for each type of visual location when comparing types of return situations. Specifically, the results for the absolute measures were *H*(1,2) = 0.95; *p* = 0.62 (total fixation number on the upper body), *H*(1,2) = 0.68; *p* = 0.71 (total fixation time on the upper body), *H*(1,2) = 3.71; *p* = 0.15 (total fixation number on the lower body), *H*(1,2) = 3.75; *p* = 0.15 (total fixation time on the lower body), *H*(1,2) = 0.04; *p* = 0.97 (total fixation number on the ball), *H*(1,2) = 0.56; *p* = 0.75 (total fixation time on the ball), *H*(1,2) = 2.08; *p* = 0.35 (total fixation number on other visual locations), and *H*(1,2) = 1.97; *p* = 0.37 (total fixation time on other visual locations). The results for the relative measures were *H*(1,2) = 0.75; *p* = 0.68 (percentage of fixation number on the upper body), *H*(1,2) = 0.83; *p* = 0.65 (percentage of fixation time on the upper body), *H*(1,2) = 3.75; *p* = 0.15 (percentage of fixation number on the lower body), *H*(1,2) = 3.21; *p* = 0.20 (percentage of fixation time on the lower body), *H*(1,2) = 3.76; *p* = 0.15 (percentage of fixation number on the ball), *H*(1,2) = 3.81; *p* = 0.14 (percentage of fixation time on the ball), *H*(1,2) = 2.07; *p* = 0.35 (percentage of fixation number on other visual locations), and *H*(1,2) = 1.97; *p* = 0.37 (percentage of fixation time on other visual locations).

## 4. Discussion

The novelty of our study was that it explored the visual behaviour displayed by expert padel players when playing different match games on court. This is the first exploratory research examining natural gaze patterns displayed by skilled athletes of this racket sport on the playing field. We specifically used a portable eye tracker to record visual fixations when returning different shots performed by their counterparts during different representative situations in padel. In doing so, the participants had the opportunity to perform natural sport-specific patterns of movements, and we were thus better able to test their perceptual–motor expertise using the dorsal ‘vision-for-action’ pathway to make online visually controlled movements [47].

Findings revealed no differences in any gaze variable between types of situations. Therefore, it seems that the players displayed a similar gaze behaviour irrespective of the returning action to perform when playing against their opponents on court. Thus, the ball-flight trajectory and the upper kinematic information from the opponents’ movements were the visual locations with more fixations and fixation time, and the percentage of fixations and fixation time, compared to the other areas of interests, when padel players returned serves, volleys, and trays/smashes from their counterparts. This visual pattern is in accordance with the study carried out by Nakamoto et al. in a virtual environment, which concluded that the kinematics from the counterparts together with the ball-flight information were the two sources of information most relevant to perceive the speed of a moving ball and estimate when and where the target will arrive for successful baseball battings [48].

A detailed inspection of the gaze behaviour for the recordings of the playing games, through the *frame-by-frame* technique, revealed that smooth pursuit tracking of the ball usually began when the ball was released from the opponent’s racket and ended at the participant’s racket–ball contact. This visual behaviour is similar to others found in previous tennis studies when expert players focused on the visual location of the ball during the return of a tennis serve [5,12,49]. For instance, Singer et al. found that the two best-ranking players tracked the ball trajectory for almost the whole duration of flight up to and following ball bounce when receiving serves [27]. Luis-del Campo et al. found that ball-flight trajectory was the visual location with the highest percentage of fixation time when tennis players, located near to the net location, had to simulate a volley for a sequence of video-projected forehand and backhand shots performed by an expert player placed at the backcourt (43.98% for experts and 38.67% for novices of total fixation time) [50]. Similarly, this ball location was most fixated on by tennis players when returning a passing-shot rally performed by an expert opponent, both in a laboratory setting (62.07% total fixation time) and on a tennis court (58.76% total fixation time) [28]. Land and McLeod also found that cricket batsmen tracked the ball for between 50% and 80% of the ball trajectory [51].

We suggest that ball trajectory information provided padel players with crucial visual information to coordinate their actions when interacting with their opponents in situ (i.e., early ball flight information was used to return opponents’ shots during game plays on court). In this vein, visual fixations on the object flight shape a common organisation of visual behaviour displayed by expert players during live-action approaches of interceptive studies such as volleyball [15,52]; or table tennis [53]. Ball flight tracking was also a beneficial hitting strategy to estimate when the target will hit and to predict the time to contact of a moving target in cricket [54].

This visual strategy of fixating on or tracking the ball once it has been hit by the counterparts could help padel players to intercept the ball while dynamically playing different time-constrained and goal-directed actions on court. We argue that padel players focused mainly on the ball location because interceptive movements that require in situ coupled responses from athletes to return different shots of opponents, such as serves, volleys, and trays/smashes, rely on this ball-flight information [55]. This prolonged fixation time on ball trajectory may finally allow players to accurately predict when and where this object will reach them [51], facilitating anticipatory behaviours and/or regulating adaptative actions to the temporal demands of playing situations. To the best of our knowledge, the ball object is a piece of information that remains constantly available for pickup from the surrounding optical array in interceptive sports, despite transformations associated with the observers’ movements and the environment. The perception of this visual information source can help padel players to better hit the ball because it directly specifies the time to contact [56].

The second visual location more fixated on in this study was the upper body because it achieved a higher percentage of fixation time than the lower body, independently of the return situation, and for the tray/smash and volley situations. Visual inspection of the sequences of play revealed that the fixation time for this area occurred during the preparatory phase of an opponent’s hitting. Similarly, Takeuchi and Inomata found that expert baseball batters moved their gaze from the proximal part of the body (head, chest, or trunk of the pitcher) to the pitching arm and the release point before the pitcher released a ball. Expert players in past studies of fast ball sports displayed a perceptual strategy relying on the precontact kinematic cues of their opponents [57]. For example, tennis players decreased the accuracy of their responses when information regarding the racket, trunk, and legs was neutralised [58], or they rarely predicted the stroke direction when the arm and upper body cues were removed [59]. Along this line, tennis players had the lowest response accuracy scores when the arm and racket condition was manipulated, implying that this distal information was relevant when attempting to anticipate an opponent’s actions with filmed sequences in stick figure format.

These evidences reinforce the assumption that expert players, compared to novices, were more sensitive to the occlusion of some advanced cues related to their counterparts’ movements when this relevant information was manipulated with different procedures of filmed sequences (e.g., point-light displays and/or display dynamics). Altogether, it seems that padel players integrated the ball-flight information and some kinematics from the upper body of the opponents to complete their interceptive actions on court.

We reasoned that past experiences in padel helped participants to develop an effective visual pattern, driving their gaze behaviours toward the relevant locations available in the sport environment. This accumulation of previous visuomotor experiences has been useful for expert athletes’ performance [60]. Specifically, the accumulation of these bodily experiences has been useful for guiding motor responses of players because (i) the exposure or familiarity to a situation or context helps them to read those cues related to opponents’ movements and object flight to anticipate their actions (*perceptual experience* hypothesis), and (ii) the capability to perform a motor skill facilitates how an athlete perceives the environment (*motor experience* hypothesis) [61]. For example, Shim et al. concluded that skilled tennis players were more accurate than novices in anticipating the type and direction of shots with video but also with live displays [22]. Experts were also faster when they had to return balls hit by a live opponent compared to returned balls projected from a ball machine.

We conclude that expert padel players used the visual information of the ball and upper kinematics from their opponents to perform different interceptive actions in situ when playing against their counterparts on court. Based on this result, the live gaze patterns of expert padel players were characterised by a prolonged tracking of ball-flight trajectory together with fixations on the trunk, shoulders, and arm–hand–racket while returning different shots performed by counterparts in “real-life” situations.

## 5. Perspectives and Future Research Lines

In future studies, it would be interesting to test whether this tendency in data would be found with large samples of participants. Future investigations should be conducted comparing visual behaviours of padel players of different skill levels, within the expert–novel paradigm, to address whether high-skilled players display a differentiated visual pattern compared to their low-skilled counterparts, focusing the gaze on the same or different visual cues available in the environment. Additionally, future studies should use other ocular metrics, such as saccadic eye movements, to explore the contribution of foveal but also peripheral vision when playing under different match–play conditions. This is a relevant issue because saccades are related to different cognitive processes (e.g., attention; see [62]), and this capacity to allocate relevant stimuli during time-constrained tasks is crucial in fast ball sports such as padel.

Future research in padel should specifically include measures at a decisional and motor level (e.g., response accuracy, reaction times, percentage of anticipatory responses) to address how information is coupled with the movement capacities of participants in the temporal course of the action (i.e., how the information perceived by the padel players constrains their actions and vice versa). For example, are anticipatory responses in padel players associated with a visual behaviour more focused on the kinematics of opponents and also on the contextual or probabilistic information of the context? Are reactive responses in these athletes more related to visual behaviours characterised by a regular tracking of ball trajectories? Is response accuracy influenced by the type of response (anticipatory vs. reactive response) displayed by players? Investigations along this line could reveal how changes in the spatiotemporal demands of tasks could elicit a different use of visual information (e.g., to respond to an opponent’s service performed in the background of the court vs. an opponent’s volley performed near to the net).

We also encourage further studies on eye movements in dynamic settings such as sports and physical education contexts because athletes and students usually perform their actions under a time-pressure condition. Eye-tracking research can provide a better understanding of how practitioners improve/learn their motor skills while solving different tasks proposed by their coaches and/or teachers. Specifically, the use of portable eye trackers may deepen our understanding of conscious and/or subconscious human behaviour (e.g., attentional focus, decision making, reasoning and/or thinking, cognitive load), offering a quantitative measure of the visual information collected by practitioners of different skill levels to control their movements during real-world scenarios.

Following attentional guidance, novices could enhance the detection of situational affordances available in their specific environments if their educators design adequately technological learning environments. For example, coaches and teachers could prepare different instructional methods with digital media (e.g., visualisations, animations, simulations) to enhance the use of those visual locations perceived by experts and associated with a more active decision-making activity and motor performance. Directing their attention to those areas with high information value, low-skilled practitioners can have the chance to understand the play, interacting adequately with teammates, counterparts, and objects available during training and/or practice.

## 6. Strengths and Limitations

This study had several strengths. First, we analysed the visual behaviours of expert padel players in real-world conditions while performing different match games. Therefore, we ensured the ecological validity measuring natural gaze patterns. The perception–action couplings of these players were also guaranteed because we collected their gaze behaviours when performing padel shots on court. To achieved this, we used a portable eye tracker that helped participants to perceive and move without any movement constraints.

The main limitation of this study was the low number of players participating in this study, and also the limited number of games played by these athletes. Expert padel players had a reduced time availability to complete large gaze data sets because the measures of visual patterns were collected within the multiple-activities schedule planned by the Regional Padel Federation for a weekend stay. This small sample of participants prevented us from testing any generalisability of the results. Therefore, our implications for practice posited in the next Practical Perspective section should be taken with caution, and they should be examined more accurately through future experimental research with a large sample of expert padel players. Additionally, the lack of a robust motion-capture system prevented us from integrating the perception and action couplings of padel players when playing on court against their counterparts. This integration between information and movement would offer a better understanding of the underlying cognitive processes supporting high levels of performance for this fast ball/racket/interceptive sport.

## 7. Practical Perspective

The ability to keep the gaze on specific areas of interest appears to be a key perceptual–cognitive skill to achieve expertise in the sport. Eye-tracking technology with wearable head-mounted systems provides sport psychologists and coaches with the opportunity to identify athletes’ gaze behaviours while training/practising/competing in situ. In this context, the data are collected in more ecological experiments because the players observe specific stimuli available in the environment and act with maximum mobility, rather than in video-based laboratory scenarios. This is a relevant question because previous studies have found differences in the visual and motor behaviours of athletes when performing in controlled laboratory settings compared to “real-life” situations [19,29]. Specifically, the portable head-mounted system utilised in this study captured the natural visual behaviours of padel players when interacting with their opponents on court. This device also ensured instant feedback of the visual behaviours displayed by players during playing situations because its software allowed live observation of their gaze recordings, facilitating the control of the study progress. Therefore, this type of study is significant for athletes and coaches because the former are interested in optimising their gaze behaviours when competing against opponents in the playing field [17].

Our data revealed some practical implications for coaches and athletes, but they should be noted with the precautions previously stated in the Strengths and Limitations section. For example, we found that the mean duration of fixation for the ball location in real-world padel games was quite long (122,305 ms/109 fixations = 1122.06 ms; see Results section). The fixation time on ball trajectory was the highest for the three returning situations in this study. Therefore, responding to the question “What needs to be seen?”, it seems reasonable that the coaches should guide the visual attention of their players towards the ball trajectory, once the opponent has hit the ball, with a statement such as “Keep your eyes on the ball” because the perception of this object would enhance the relationship between players and the performance environment [56].

Taking into consideration that focusing on the ball was also relevant for previous studies in tennis carried out in on-court situations [27,28], this recommendation of fixating for a long time on this visual location could also be generalised for other interceptive sports because the perception of the ball-flight trajectory directly specifies spatial and temporal information to accurately hit the ball in a changing performer–environment system (e.g., players could use the information of an approaching ball to visually adapt movements in the playing field). As players observe different ball speeds during competitions, coaches could manipulate the velocity of the balls thrown to their players during training (e.g., increasing or decreasing ball speed in training tasks; see [63,64]. Meanwhile, the players should calibrate the available time to complete their actions (e.g., varying their movements to the specific information of the ball features). Regarding the possible transfer effect of practising one interceptive sport on the motor learning of another interceptive sport, our opinion is that a positive transfer could occur if these sports share similar movement patterns, the players are familiar with the perception of ball trajectories, and they have accumulated experience in using an implement such as a racket in hitting tasks. However, these observations should be addressed with data from further experiments.

A further particular challenge for the use of eye-tracking systems is the algorithmic analysis of gaze data (i.e., the algorithmic gaze–cue allocation of large amounts of raw gaze data; see [18]. However, the advances in head-mounted eye trackers have made new performance-focused interventions possible—for example, the design of vision-based training programmes [65]. Along this line, researchers should redirect the visual attention of low-skilled padel players towards the opponents’ upper body and ball trajectory because these two areas of interest were the most fixated on by expert players to perform interceptive actions when interacting with their counterparts on a padel court. We argue that perceptual training for this sport should promote an attentional strategy with foveal vision to some upper kinematics of opponents’ movements (e.g., shoulders, trunk, and the region of arm–hand–racket) and ball-flight information through the use of explicit and/or implicit methods. Therefore, padel coaches should guide the eyes of their novice players to these relevant visual locations in order to facilitate accurate interceptions of the ball (e.g., returns of the counterparts’ shots) when playing in on-court situations. As a result, coaches should promote directing players’ attention to features in these areas during their interventions in an attempt to predict opponents’ intentions.

The focus of attention is an influencing factor for the perception–action couplings of interceptive sports. Along this line, education regarding attention is very relevant during the performance of time-constrained tasks (e.g., guiding the attention to more specific information for controlling movements; see [66]. Therefore, how could padel coaches manage to drive the visual attention of their players towards these relevant stimuli? For example, they could achieve this by (i) providing initial instructions to the players about where they should fixate (“Keep your eyes on the shoulders, or trunk, or the region of arm–hand–racket” and “keep your eyes on the trajectory of the ball once the opponent hits it”) to calibrate what combination of these areas of interest would be more useful to achieve successful returns from the opponents, and (ii) manipulating different sources of contextual information available during the training tasks (e.g., the orientation of the counterparts’ shoulders or the position of their arms–hands–rackets when they hit the ball to improve judgments about ball directions).

Altogether, the exploration of expert padel players’ gaze patterns during on-court situations would offer sport psychologists and coaches the chance to improve the understanding of perceptual–cognitive processing involved in athletic performance [17,63]. This information is needed to correctly plan training tasks in this racket sport. In this context, the players could experience the visual demands existing in real matches of competitions when performing tasks with high time pressure.

## Data Availability

The data presented in this study are available within the article (see tables), and the datasets analysed in this study are available on request from the corresponding author.

## Supplementary Materials

The following supporting information can be downloaded at: https://www.mdpi.com/article/10.3390/s23031438/s1, Video S1: smash-tray return. Video S2: serve returns. Video S3: volley returns.
